# Short-chain fatty acids differentially regulate feed intake and hypothalamic appetite-related genes in layer-type chicks

**DOI:** 10.3389/fphys.2026.1825952

**Published:** 2026-09-16

**Authors:** Elham Ghashghaei, Ahmed Mijiyawa, Minghui Wang, Morteza Zendehdel, Hai Lin

**Affiliations:** 1 Key Laboratory of Efficient Utilization of Non-grain Feed Resources (Co-construction by Ministry and Province), Ministry of Agriculture and Rural Affairs, Shandong Provincial Key Laboratory of Animal Nutrition and Efficient Feeding, Department of Animal Science, Shandong Agricultural University, Taian, Shandong, China; 2 Regional Center of Excellence for Avian Science, University of Lome, Lomé, Togo; 3 Department of Basic Sciences, Faculty of Veterinary Medicine, University of Tehran, Tehran, Iran

**Keywords:** feed intake, hypothalamus, ICV injection, layer-type chicks, sodium acetate, sodium butyrate, sodium propionate

## Abstract

**Introduction:**

Activation of hypothalamic neurons by short-chain fatty acids (SCFAs) has been recognized as one of the mechanisms of appetite regulation in mammals. However, their central effects on hypothalamic neuropeptides have not yet been fully investigated in chicks. In the present study, feed intake and mRNA expression of neuropeptides in chicks were evaluated under the influence of SCFAs.

**Methods:**

The experiments included intracerebroventricular (ICV) injection of SCFAs into five-day-old chicks. Feed intake was recorded up to 2 h post-injection, and PCR was used to determine the gene expression levels of neuropeptides.

**Results:**

Feed intake significantly increased following ICV administration of sodium acetate (0.25 mg) and sodium butyrate (0.5 mg) (P < 0.05), whereas it was reduced by ICV administration of sodium propionate (5 mg) (P < 0.05). NPY5R mRNA expression significantly increased by sodium acetate (0.25 mg) (P < 0.05); however, NPY, NPY4R, AgRP, and anorexigenic neuropeptides were not altered (P > 0.05). NPY mRNA expression was significantly increased by sodium butyrate (0.5 mg), whereas reductions in POMC and MC4R were observed following this treatment (P < 0.05); however, AgRP, NPY4R, and MC1R were not affected (P > 0.05). An increase in MC4R mRNA expression was induced by sodium propionate (5 mg) (P < 0.05), while no significant changes were detected in orexigenic neuropeptides or POMC and MC1R (P > 0.05).

**Conclusion:**

Our findings suggest that SCFAs regulate feed intake in chicks by acting in the hypothalamus.

## 1 Introduction

The regulation of appetite is a significant biological function that regulates growth, reproductive performance, and the ability to produce food efficiently through the integration of signals received from both the brain and the peripheral nervous system (Camilleri, 2015; Ueno and Nakazato, 2016). As global food supply concerns continue to increase the demand for poultry meat and eggs (Vlaicu et al., 2024), understanding the key factors that regulate feed intake in broiler chickens and body weight maintenance in layer hens has become increasingly important (Richards and Proszkowiec-Weglarz, 2007; Cao et al., 2024; Saneyasu, 2024). Better insight into energy homeostasis and the neuromolecular pathways that direct appetite regulation may improve productivity within the poultry industry (Ghashghaei et al., 2025a).

The neurons located in the arcuate nucleus (ARC) of the hypothalamus regulate eating behavior and maintaining energy balance (Fu, 2025). Neuropeptide Y (NPY) and agouti-related peptide (AgRP) are orexigenic neuropeptides that promote hunger (Chen Y. et al., 2016; Mahdavi et al., 2024), whereas pro-opiomelanocortin (POMC) and cocaineand amphetamine-regulated transcript (CART) promote satiety (Lenard and Berthoud, 2008; Hill, 2010). The hypothalamus processes and integrates signals from nutrients and hormones to assess the body’s energy status (Blouet and Schwartz, 2010).

Short-chain fatty acids (SCFAs), which result from the digestion of food products by intestinal bacteria, influence the hypothalamus and affect appetite (Frost et al., 2014). Acetate, a type of SCFA, can cross the blood–brain barrier through the action of specific fatty acid transport proteins (Mitchell et al., 2011) and stimulate the activity of hypothalamic neurons (Frost et al., 2014). Intravenous acetate administration increases POMC and decreases AgRP gene expression, thereby reducing feed consumption in mice (Frost et al., 2014). The mechanism of acetate’s effects may involve AMP kinase (AMPK) and acyl-CoA carboxylase (ACC) (Frost et al., 2014), and acetate reduces feed consumption through its action on orexin/hypocretin neurons in the hypothalamus (Forte et al., 2024) primarily through gut–brain axis signaling involving neural, endocrine, and metabolic pathways.

Butyrate reduces feed consumption by inhibiting the activity of appetite-stimulating neurons in the hypothalamus, and propionate reduces feed intake and influences hypothalamic neuropeptide expression (Li et al., 2017; Jiao et al., 2020; Zhang et al., 2022). SCFAs are involved in fat metabolism, appetite regulation, and maintenance of energy balance through gut–brain neuronal pathways (Vadder et al., 2014; Byrne et al., 2015).

Most research has been conducted in mammals, and physiological and neuroendocrine differences limit the direct application of these findings to poultry (Newmyer et al., 2013; Ramser and Dridi, 2022). Therefore, studying feed consumption directly in avian species is necessary.

SCFAs may promote intestinal development throughout the entire developmental period of the broiler (Liao et al., 2020) and may represent an important mechanism by which SCFAs help mitigate necrotic enteritis in poultry by modulating the immune response (Gómez-Osorio et al., 2021). SCFAs improve egg quality, including shell hardness and the amount of dirt, cracks, and deformities on the surface. Layer chicks that have been fed SCFAs before they lay their first egg also experience a greater hatching success rate than those that do not (Sengor et al., 2007). SCFA supplements also improve fat content, in terms of total cholesterol and abdominal fat, in chickens raised for meat production (Khatibjoo et al., 2018). These findings indicate that SCFAs influence multiple aspects of poultry production.

However, the effects of SCFAs delivered directly to the central nervous system (CNS) in chicks remain unknown. Although the effects of SCFAs with intravenous and oral administration have been studied, little is known about their effects after central injection. To date, no studies have directly examined the central effects of SCFAs on feed intake control and hypothalamic appetite-related neuropeptide expression in avian species.

Acetate, butyrate, and propionate were selected in our study due to their different roles in gut–brain signaling and potential differential effects on hypothalamic neurons. Layer-type chicks were chosen because the growth phase is important in determining egg production potential. Laying hens that do not achieve an optimal weight will lay fewer and smaller eggs, whereas laying hens with excessive weight will consume more feed, store excess fat, and show higher mortality rates (Pérez-Bonilla et al., 2012; Lu et al., 2023). Therefore, understanding how feed consumption is controlled in the central nervous system is important for maintaining optimal weight and improving performance.

This study investigated the effects of intracerebroventricular (ICV) administration of sodium acetate, sodium butyrate, and sodium propionate on feed intake control. To preferentially assess central actions of SCFAs, ICV administration was administered to avoid gastrointestinal absorption. Yet, because of bidirectional communication between the brain and peripheral organs, this rules out downstream peripheral responses following central administration. We hypothesized that central SCFA injections influence feed intake and neuropeptide expression in chicks.

## 2 Materials and methods

### 2.1 Experimental animals

In five separate trials, the impact of three different sodium salts (sodium acetate, sodium butyrate, and sodium propionate) on both feeding behavior and the expression, at the mRNA level, of appetite-regulating neuropeptides in layer-type chickens was studied.

To minimize biological variation with respect to sex-related differences in the neonatal period, male layer-type chicks were selected. Although male and female chicks have comparable feeding-related physiological traits at this early developmental age, males were used to avoid the confounding influence of ovarian development and reproductive-endocrine changes that occur later in life.

A total of 144 one-day-old male layer-type chicks were sourced from a commercial hatchery located in Tai’an, China, and were housed in groups for two days before being placed individually into cages kept at a temperature of 32 °C and 50% relative humidity. During the experimental period, individual housing facilitated accurate feed consumption from each chick and prevented social interactions in groups that could affect interindividual feed intake differences in this trial. Throughout the trial, all neonatal laying chicks were fed a layer-type starter mash (**Table 1**) and were given unlimited access to water according to our previous studies (Zhao et al., 2009; Ghashghaei et al., 2025c). At five days of age, the chicks received brain administration of the three sodium salts of fatty acids. The present study received approval from the Animal Care Committee of Shandong Agricultural University, China, for the use of experimental chickens.

**Table 1:** Ingredient composition and nutrient content of the basal starter diet for layer-type chicks.

Item	Starter phase
Ingredients, %	
Corn (8.3% CP)	50.50
Soybean meal (43% CP)	33.00
Corn gluten meal (60% CP)	4.00
Wheat middling	5.00
Soybean oil	2.00
Salt	0.28
Limestone	1.75
Monodicalcium phosphate	1.55
Choline chloride	0.10
L-Lys·HCl (99%)	0.80
DL-Met (98%)	0.35
L-Thr (98%)	0.29
Probiotics + Enzyme	0.13
Vitamin premix^1^	0.05
Trace mineral premix^2^	0.20
Total	100.0
Nutrient composition	
Crude protein, %^4^	19.98
ME, kcal/kg^3^	2924
Total Ca, %^4^	0.88
Non-phytate P, %^3^	0.50
Lys, %^4^	1.25
Met, %	0.51
Met + Cys, %^3^	1.070
Thr, %^4^	0.68
Trp, %^3^	0.283

^1^ The vitamin premix provides the following quantities per kilogram of diet: vitamin A, 8000 IU; vitamin D3, 1000 IU; vitamin K, 0.5 mg; vitamin E, 20 IU; vitamin B12, 0,01 mg; cholecalciferol, 1300 IU; riboflavin 8 mg; niacin, 35 mg; pantothenic acid, 10 mg; biotin, 0.18 mg; folic acid, 0.55 mg.

^2^ The mineral premix provides the following quantities per kilogram of diet: Fe, 100 mg; selenium, 0.3 mg; copper, 8 mg; Zinc, 100 mg; I, 0.7 mg; manganese, 120 mg.

^3^ Nutrient composition calculated.

^4^ Nutrient composition measured.

### 2.2 SCFAs

This research employed sodium acetate (CH_3_COONa · 3H_2_O, MW 136.08; Tianjin Kaitong Co., Ltd.; China), sodium butyrate (C_4_H_7_NaO_2_, MW 110.09; Aladdin Industrial Corporation; Shanghai, China), sodium propionate (CH_3_CH_2_COONa, MW 96.06; Sigma-Aldrich), and Evans Blue (C_34_H_24_N_6_Na_4_O_14_S_4_, MW 960.81; SAIT). First, the SCFAs were dissolved in saline and then combined with Evans Blue saline solution. A control solution was prepared using the same saline and Evans Blue components (Ghashghaei et al., 2025a) without SCFAs. The treatment and control groups received an injection volume of 10 μL (Ghashghayi et al., 2022).

### 2.3 Method for ICV injection

Layer-type chicks were first weighed before being divided into groups of eight. A single ICV injection, delivered without anesthesia, was administered to each 5-day-old chick using a Hamilton microsyringe (Davis et al., 1979; Furuse et al., 1997). To avoid the effects of anesthetic agents on hypothalamic function and feeding behavior, ICV injections were performed without anesthesia, as reported in previous studies (Davis et al., 1979; Furuse et al., 1997). While handling and restraint may cause a temporary stress response, the ICV procedure conducted in neonatal chicks is quick (overall less than 10 min) and has been widely used in previous studies. The time of restraint was kept to a minimum for stress-related effects. Furthermore, we refrained from anesthesia as anesthetic agents can affect central nervous system (CNS) activity and feed-responsive circuits potentially complicating the interpretation of acute feeding responses.

Correct positioning of the calvarium during injection was ensured by placing the chick’s head in an acrylic holder and securing it with a 45-degree clamp (Van Tienhoven and Juhász, 1962), thereby minimizing physiological stress (Furuse et al., 1997). In this method, the time the chick is handled or restrained is minimal, and it allows rapid administration; therefore, this procedure has been widely used in neonatal chicks. Each group received the assigned injections, and only chicks whose brains exhibited blue staining after the injection were included in the data analysis (Badri et al., 2025; Zarei et al., 2025). Since all chicks in our study had Evans Blue in the anatomical landmarks of the hypothalamus, no animals were excluded from the study, and Evans Blue dye was used to visually confirm accurate ICV placement. Within 2 hours of ICV administration, brain tissues were collected from each group immediately following a meal (Ghashghaei et al., 2025a). When the experiment was finished, the chicks were euthanized by a method called cervical dislocation, which was approved by the animal care team. Right away, the tissues from the hypothalamus area of the brain were taken out, frozen quickly in liquid nitrogen, and kept at a very cold temperature, -80 °C, until it was time to extract the RNA.

Hypothalamic tissues from control birds and from treatment groups showing significant effects on feed intake were selected for gene expression analysis to examine neuropeptide responses associated with the observed behavioral changes, consistent with our previous experimental approach (Ghashghaei et al., 2025b). To minimize selection bias, chicks were randomly assigned to treatment groups. To ensure accuracy and reproducibility, all injections were performed using consistent anatomical landmarks and standardized procedures for all chicks. To reduce possible bias during data processing, brain samples were coded before analysis, assigning each sample a unique identification code rather than group labels.

### 2.4 Feeding experiments

Sodium acetate and sodium butyrate were utilized in the first and second experiments. In both experiments, four groups were created, and each group contained eight chicks. A control group was included in both experiments, along with three treatment groups (0.25, 0.5, and 1 μg). In the third experiment, sodium propionate was injected at doses of 0.25, 0.50, and 1 μg, and no effective dose was identified. In the fourth experiment, sodium propionate was administered at 2 and 3 μg, and likewise, no effective dose was detected. In the fifth experiment, doses of 4 and 5 μg were administered. Because the lower doses of sodium propionate did not produce detectable changes in feed intake, additional experiments were conducted with progressively higher doses to identify one that affected feeding behavior. The selected doses used for central injection were based on data available from previous studies (Ghashghayi et al., 2022; Safikhani et al., 2023). During each experiment, neonatal laying chicks were kept in individual cages with free access to food and water. Each cage was equipped with a drinker and a single small feed trough (Ghashghaei et al., 2025b). Feed was removed for three hours prior to injection to standardize conditions for all chickens (Zendehdel et al., 2016). The chicks were weighed before central injection. After weighing, brain injections were administered, and the chicks were returned to their feed. Feed intake was recorded at 30 minutes, 1 hour, and 2 hours after brain injection. Following each measurement, feed was removed, weighed, and promptly returned (Zendehdel et al., 2017). Two hours post-injection, injection sites were confirmed using Evans Blue dye. Only chicks with visible dye in the brain were included in the statistical analysis (Ghashghaei et al., 2025b). The results indicated that Evans Blue was visible in the target brain area of all chickens used; therefore, no samples were excluded from the data analysis. Furthermore, feed consumption at each time interval was evaluated relative to body weight, based on methods reported in previous studies (Zendehdel et al., 2016; Zendehdel et al., 2017; Ghashghayi et al., 2022; Ghashghaei et al., 2025b).

### 2.5 Extraction of RNA and quantification by RT-PCR

Total RNA was extracted from hypothalamic tissues using TRIzol reagent (Invitrogen-San Diego, CA). RNA quality was evaluated using spectrophotometry (Eppendorf, Germany) (Ghashghaei et al., 2025b). After confirming RNA quality, cDNA synthesis was immediately performed using a commercial cDNA synthesis kit (Roche, Switzerland) (Ghashghaei et al., 2025a). For real-time PCR, primers (**Table 2**) were generated using known sequences and NCBI Primer BLAST to ensure specificity for chicken gene sequences. Primer specificity was verified by melt curve analysis, which showed a single peak for each target gene, indicating specific amplification. cDNA was amplified using FastStart Universal SYBR Green Master (ROX) (Roche, Switzerland) (Ghashghaei et al., 2025a). GAPDH was used as the internal control gene to normalize hypothalamic gene expression data (Ghashghaei et al., 2025a). GAPDH is a suitable internal control for hypothalamic tissue and is stably expressed in the hypothalamus under the experimental conditions of the present study. Relative gene expression levels were calculated using the comparative CT method (2 − ΔΔCT).

**Table 2:** Primers for the targeted and reference transcripts.

Gene	Primer sequence
NPY5R	F: TGATCGGTGGATGTTTGGCA R: AGCCAACGGCCCAAATGATA
MC1R	F: CGCCACATGGACAATGTCAT R: GGTAGCGCAGCGCATAGAA
NPY4R	F: CCTGCCCTTTCTGACCACAT R: GGGATGCAGTATTGCAGAAGC
NPY	F: GAGGCACTACATCAACCTCATCAC R: TGTTTTCTGTGCTTTCCCTCAA
MC4R	F: ACACTCCAGCCTCTCCATTTCT R: TGTTCATAGCAGCCTCCCGA
AgRP	F: GGAACCGCAGGCATTGTC R: GTAGCAGAAGGCGTTGAAGAA
POMC	F: CGCTACGGCGGCTTCA R: TCTTGTAGGCGCTTTTGACGAT
GAPDH	F:ACATGGCATCCAAGGAGTGAG R:GGGGAGACAGAAGGGAACAGA

### 2.6 Statistical analysis

Feed intake data were analyzed using two-way repeated-measures ANOVA in GraphPad Prism 8.0.2. Feed intake was measured repeatedly for each chick at 30, 60, and 120 minutes post-injection; therefore, time was treated as a repeated factor in the analysis. Treatment (SCFA dose) and time were considered as main factors, and interactions between them were examined. Each laying chick served as an individual replicate for both SCFA dose and time, and main effects and interactions were examined. Multiple comparisons among groups were evaluated using the Tukey–Kramer test, and differences were considered statistically significant at P < 0.05. For hypothalamic gene expression analysis, data were analyzed using t-tests or non-parametric methods, and results were reported as mean ± SEM, since gene expression was analyzed at a single time point. Because multiple genes were tested and no correction for multiple comparisons was applied, interpretation of gene expression differences was made cautiously. Statistical significance was established at P < 0.05 in all cases.

## 3 Results

### 3.1 Central effects of SCFAs on feed intake of layer-type chicks

In Experiments 1 and 2, the central effects of sodium acetate and sodium butyrate on feed consumption were investigated at three dose levels (0.25, 0.5, and 1 µg). Feed intake was monitored at 30, 60, and 120 minutes after injection. In Experiment 1, sodium acetate at a dose of 0.25 µg increased feed consumption at 60 and 120 minutes after injection (P < 0.05), but not at 30 minutes *(P > 0.05)*. The doses of sodium acetate at 0.5 and 1 µg did not produce a statistically significant change in feed intake at any time point (*P > 0.05*; **Figure 1**). In Experiment 2, sodium butyrate at 0.5 µg increased feed consumption at 30, 60, and 120 minutes after injection (*P < 0.05)*. In contrast, sodium butyrate at doses of 0.25 and 1 µg did not significantly alter feed intake at any time point (*P > 0.05*; **Figure 2**). The effects of sodium propionate on feed intake (0.25, 0.5, and 1 µg as well as 2, 3, 4, and 5 µg) were evaluated in Experiments 3, 4, and 5, with measurements taken at 30, 60, and 120 minutes after injection. In Experiment 3, no differences in feed intake were observed at any time point following administration of 0.25, 0.5, or 1 µg sodium propionate (*P > 0.05*; **Figure 3**). Similarly, in Experiment 4, sodium propionate at doses of 2 and 3 µg did not significantly affect feed intake (*P > 0.05*; **Figure 4**). However, in Experiment 5, sodium propionate at 5 µg significantly decreased feed intake at 60 and 120 minutes *(P < 0.05)* but did not affect feed intake at 30 minutes (*P > 0.05*; **Figure 5**).

**Figure 1:**
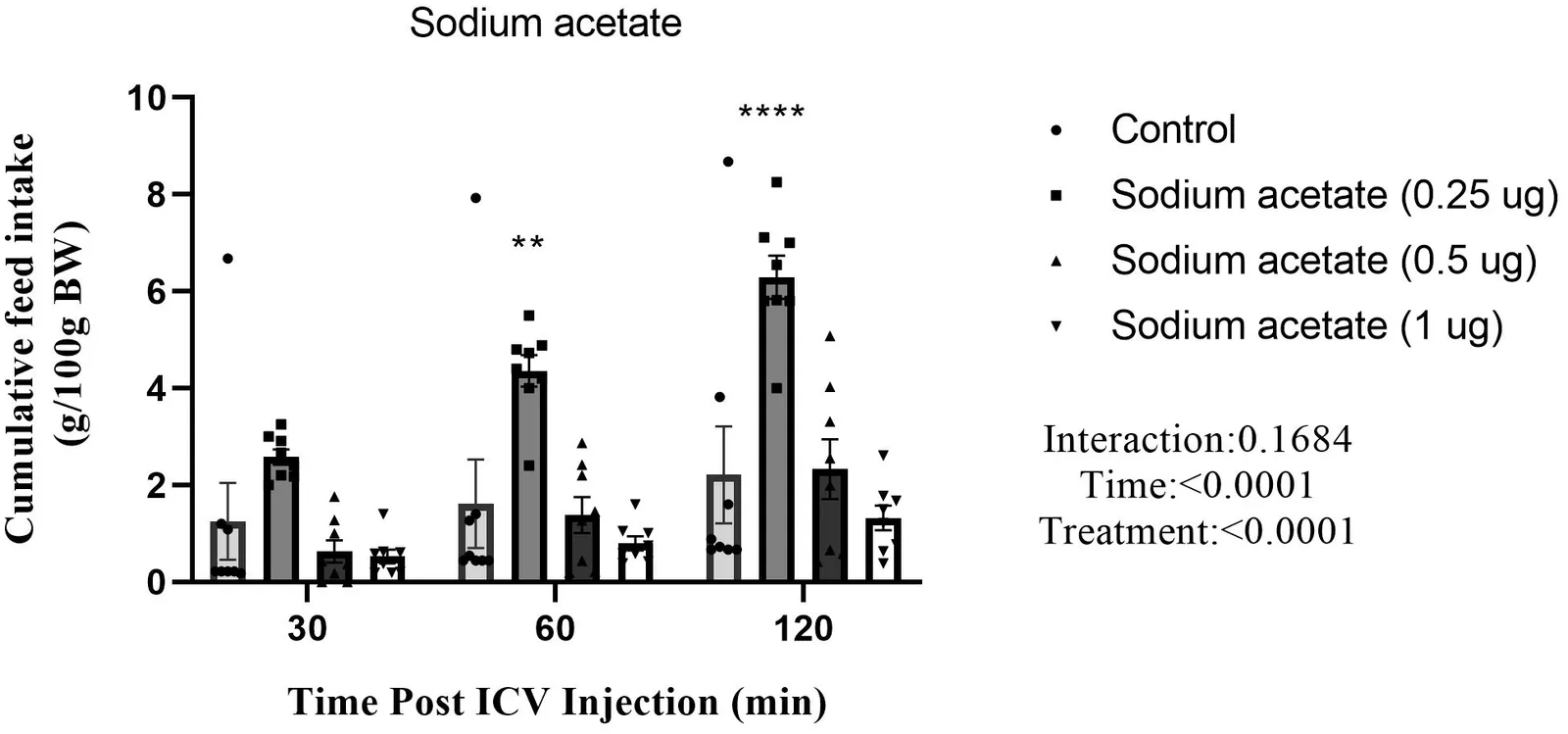
The impact of centrally administered sodium acetate at doses of 0.25, 0.5, and 1 μg on the feed intake of layer-type chicks. Measurements of feed intake were taken at 30, 60, and 120 minutes following central injection. Values are reported as mean ± SEM (n = 8). *Significant differences are indicated by **P < 0.0027 and ****P < 0.0001.

**Figure 2:**
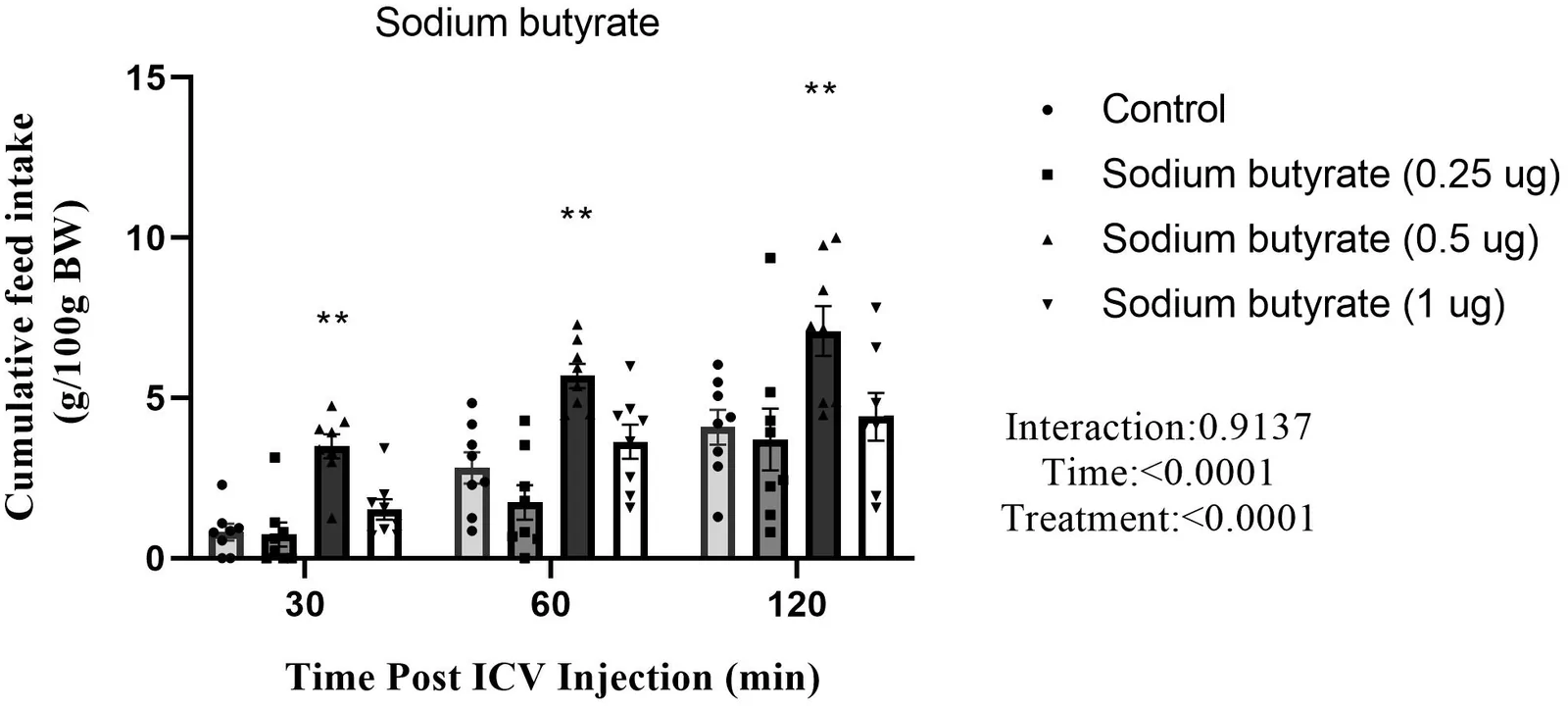
Effects of centrally administered sodium butyrate (0.25, 0.5, and 1 μg) on feed intake in layer-type chicks. Measurements of feed intake were taken at 30, 60, and 120 minutes after injection. Values are reported as mean ± SEM (n = 8). *Significant differences are indicated by **P < 0.0063, *0.0029*, *0.0016*.

**Figure 3:**
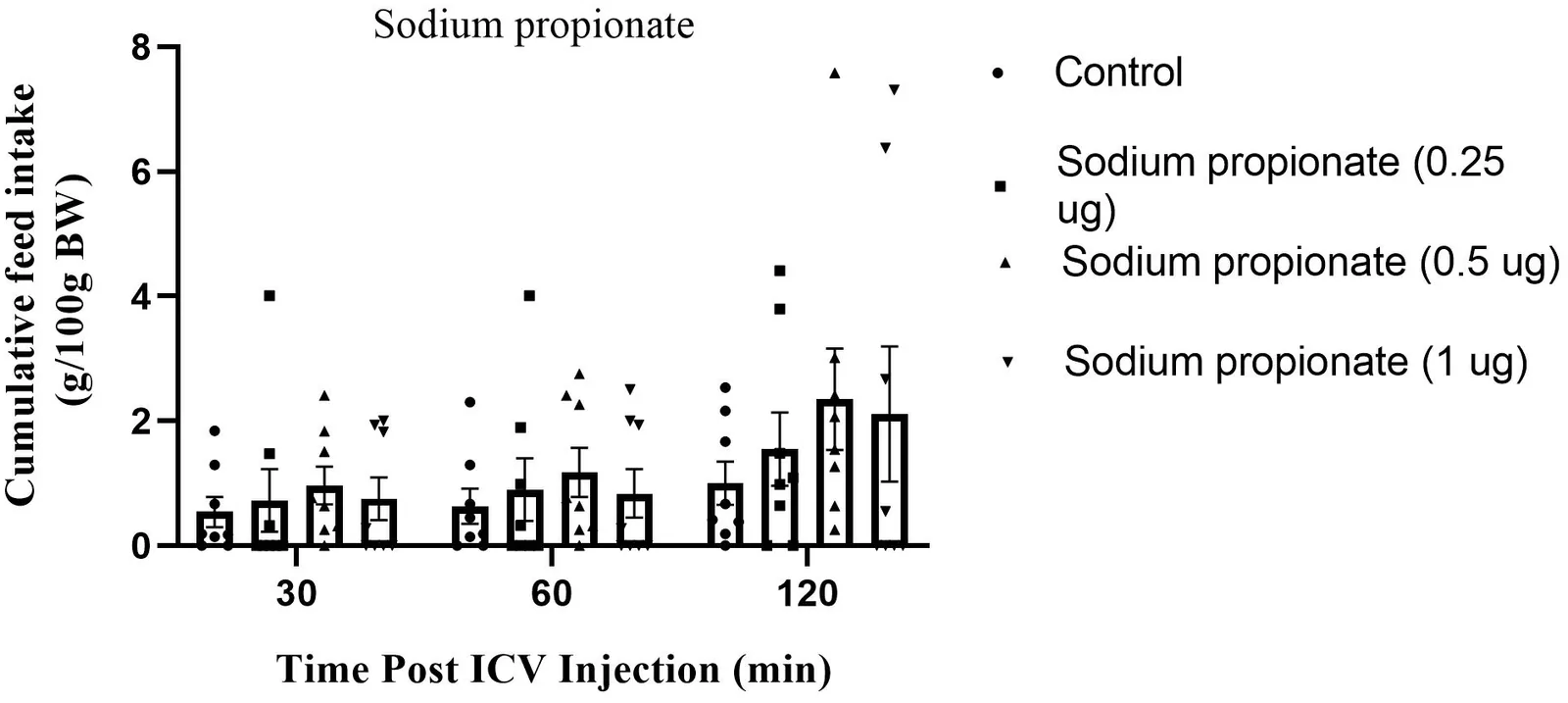
Effects of centrally administered sodium propionate (0.25, 0.5, and 1 μg) on feed intake in layer-type chicks. Measurements of feed intake were taken at 30, 60, and 120 minutes after injection. Values are reported as mean ± SEM (n = 8). No significant differences were observed compared with the control group.

**Figure 4:**
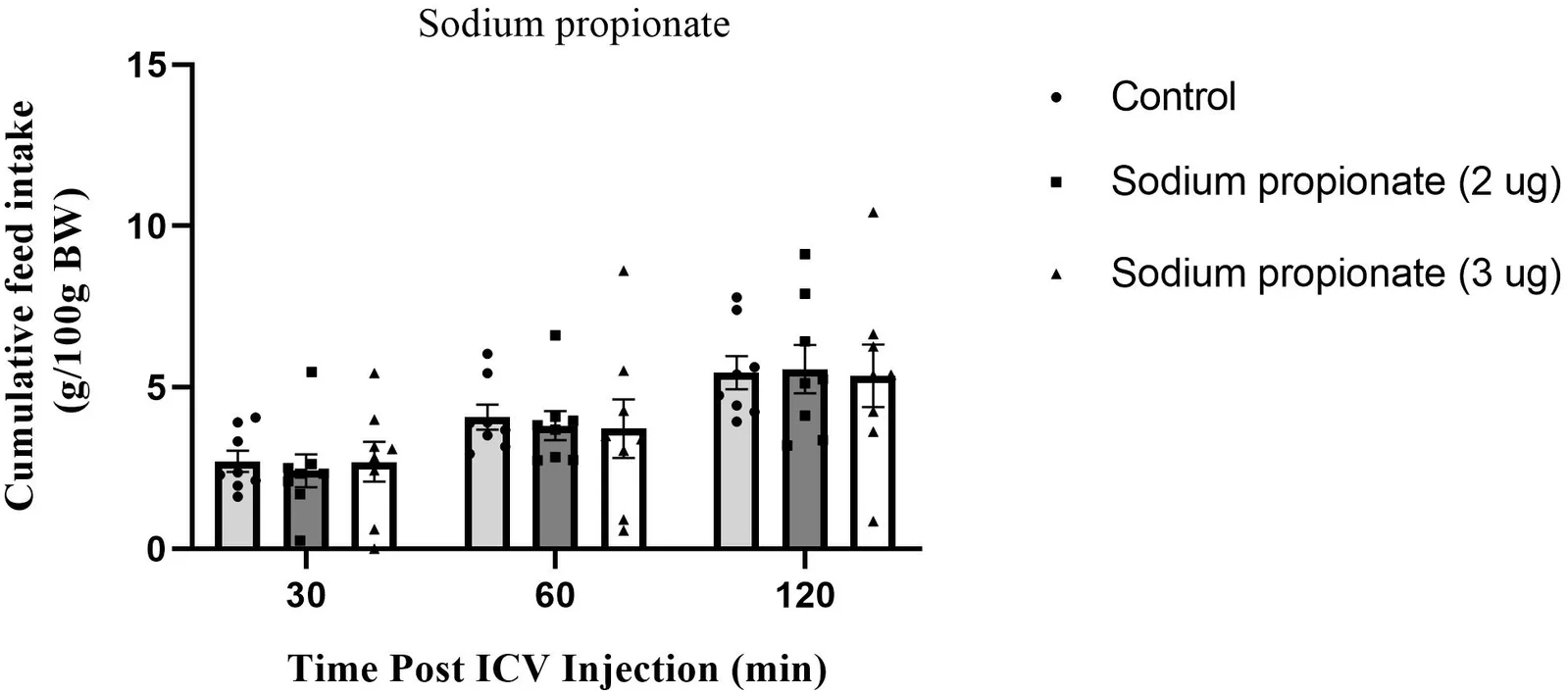
Effects of centrally administered sodium propionate (2 and 3 μg) on feed intake in layer-type chicks. Measurements were conducted at 30, 60, and 120 following central injection. Values are reported as mean ± SEM (n = 8). No significant differences were observed compared with the control group.

**Figure 5:**
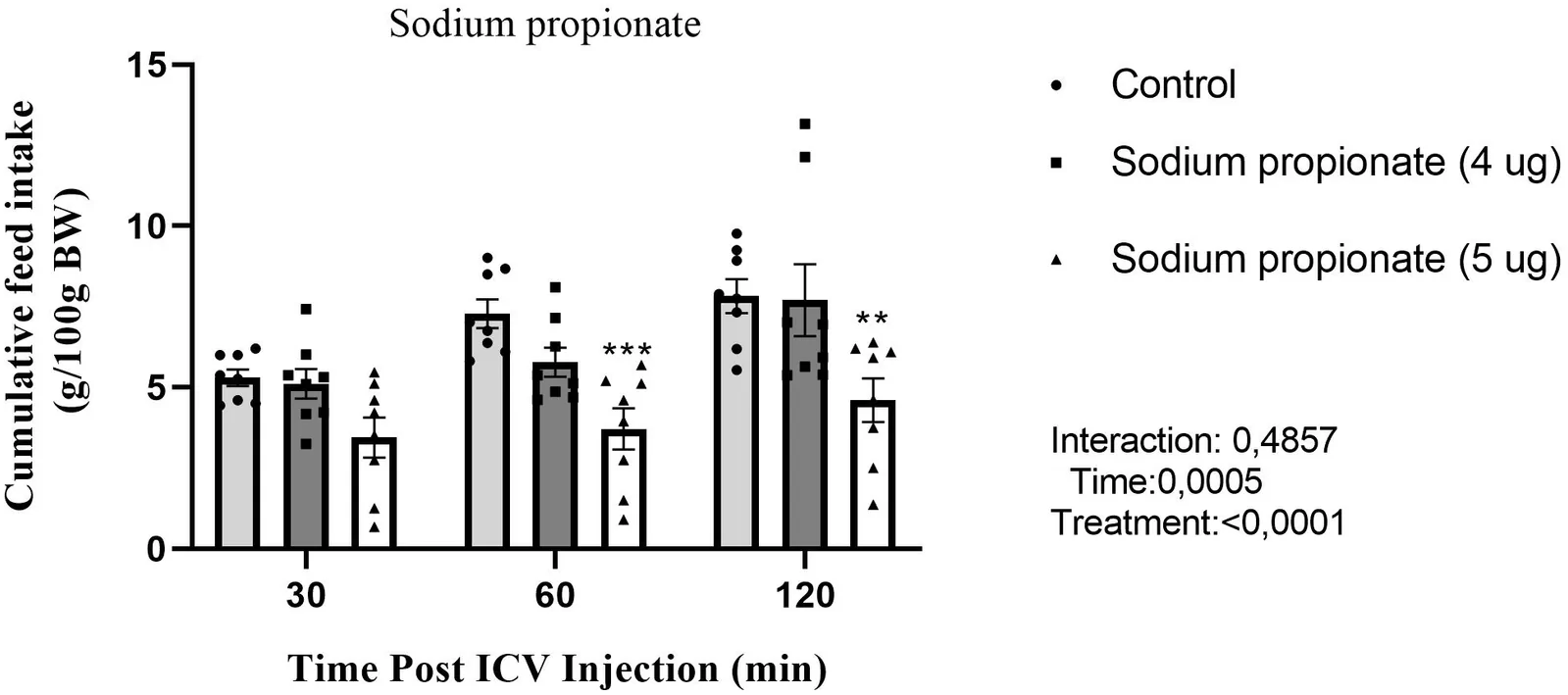
Effects of centrally administered sodium propionate (4 and 5 μg) on feed intake in layer-type chicks. Feed intake was assessed at 30, 60, and 120 minutes after injection. Values are reported as mean ± SEM (n = 8). *Significant differences are indicated by ***P < 0.0004 and **P < 0.0013.

### 3.2 Central effects of SCFAs on mRNA expression of appetite-related neuropeptides

ICV administration of sodium acetate (0.25 µg) significantly increased mRNA expression of neuropeptide Y receptor 5 (*NPY5R*) *(P < 0.05)*. However, sodium acetate did not significantly affect mRNA expression of *NPY*, neuropeptide Y receptor 4 (*NPY4R*), *AgRP*, or anorexigenic neuropeptides (*P > 0.05*; **Figure 6**). Sodium butyrate (0.5 µg) significantly reduced melanocortin receptor 4 (*MC4R*) and *POMC* mRNA expression *(P < 0.05)* but did not significantly change melanocortin receptor 1 (*MC1R*) mRNA expression (*P > 0.05*; **Figure 7**). Sodium butyrate also significantly increased *NPY* mRNA expression *(P < 0.05)*, while having no significant effect on other orexigenic genes, including *AgRP*, *NPY4R*, and *NPY5R* (*P > 0.05*; **Figure 7**). ICV administration of sodium propionate (5 µg) significantly increased MC4R mRNA expression (*P < 0.05*) but did not significantly affect mRNA expression of *POMC*, *MC1R*, or orexigenic neuropeptides (*P > 0.05*; **Figure 8**).

**Figure 6:**
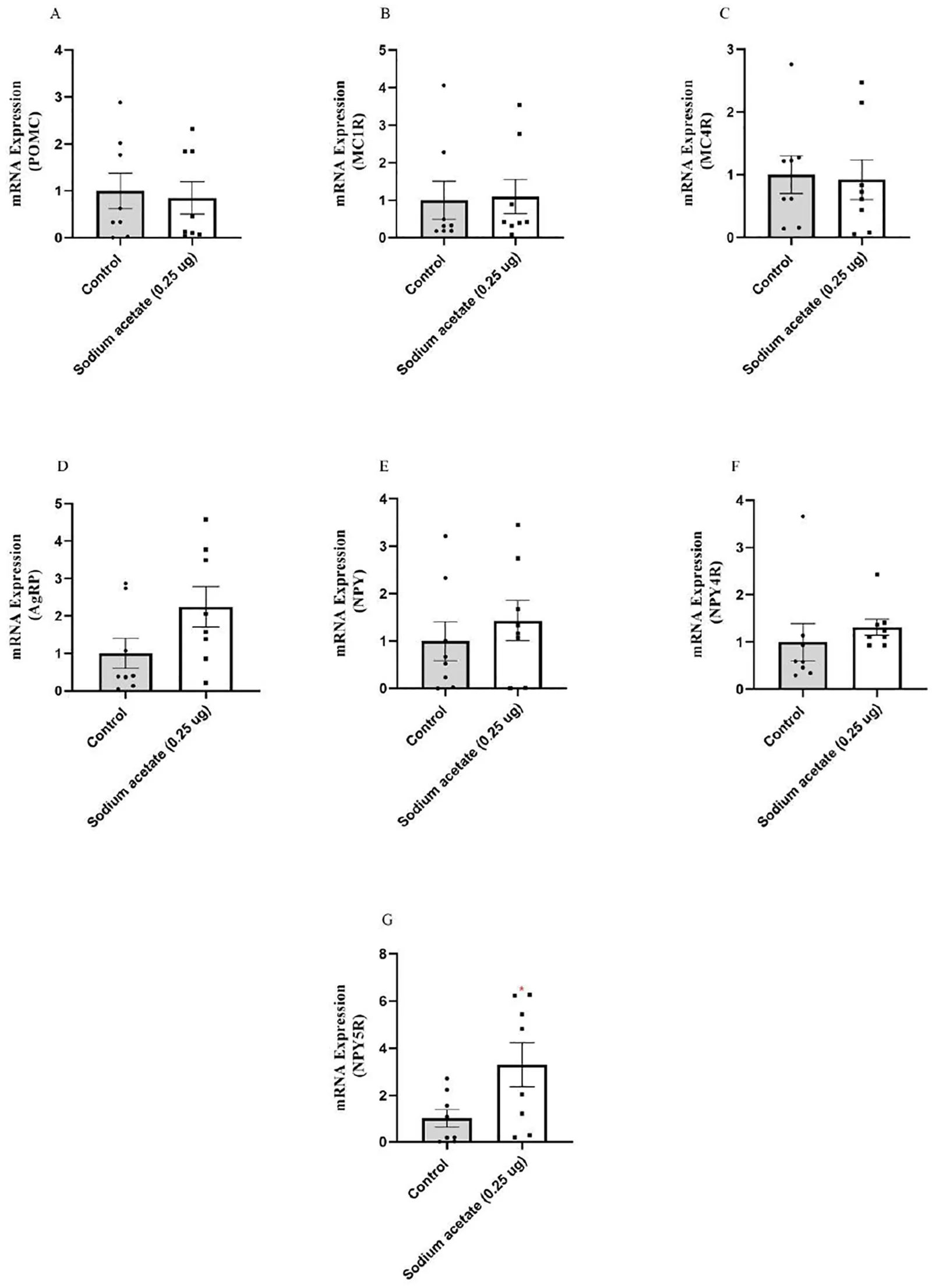
The impact of centrally delivered sodium acetate at doses of 0.25 mg on the expression of genes involved in appetite was examined in chicks. Values are reported as mean ± SEM (n = 8). *Significant differences are indicated by *P < 0.0387. The following abbreviations are used throughout the figure: **(A)** POMC (pro-opiomelanocortin). **(B)** MC1R (melanocortin receptor 1). **(C)** MC4R (melanocortin receptor 4). **(D)** AgRP (agouti-related peptide). **(E)** NPY (neuropeptide Y). **(F)** and NPY4R (neuropeptide Y receptor 4). **(G)** NPY5R (neuropeptide Y receptor 5).

**Figure 7:**
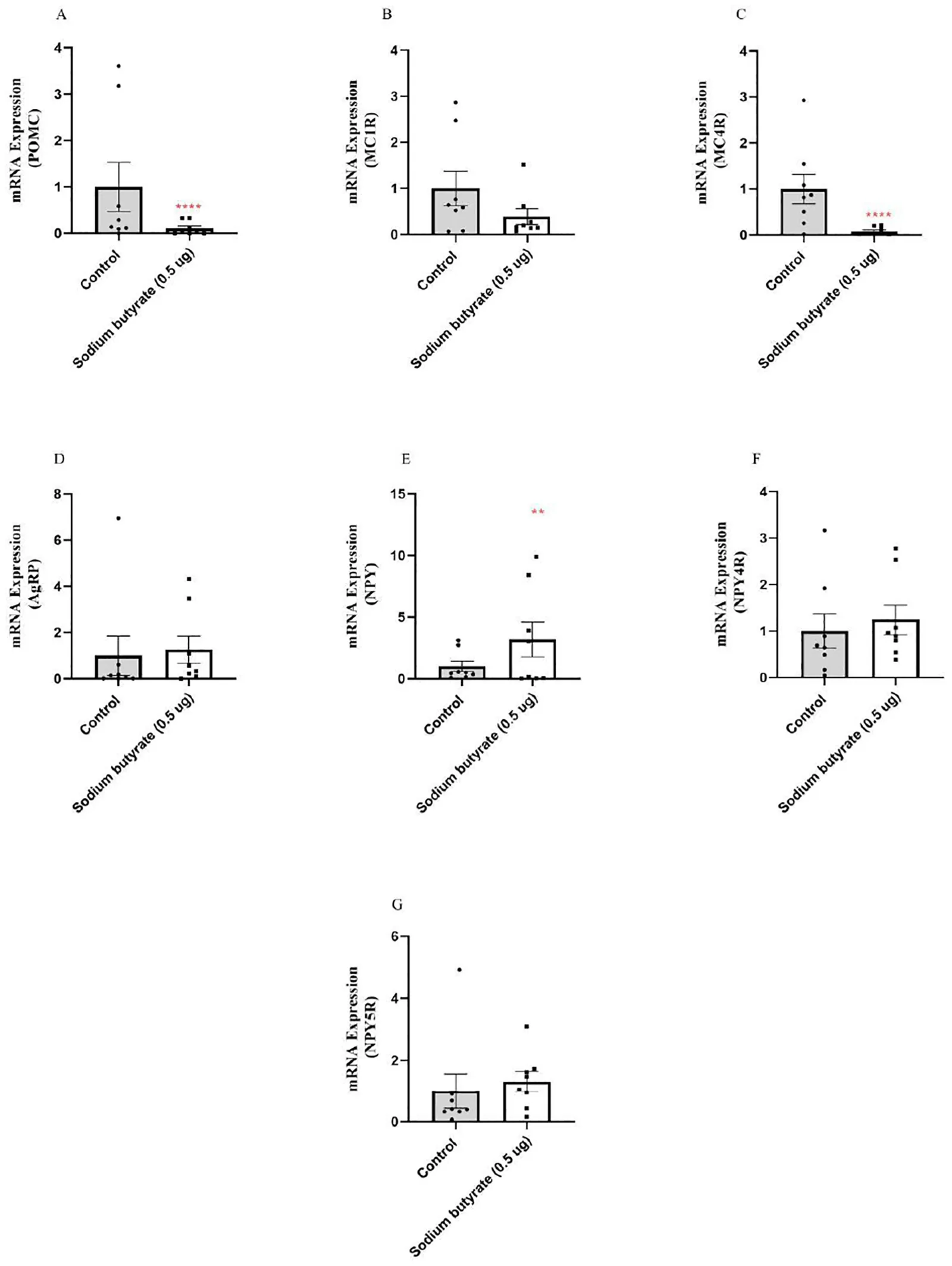
The impact of centrally delivered sodium butyrate (0.5 mg) on the expression of hypothalamic genes involved in appetite regulation was examined in neonatal layer-type chicks. The data are reported as mean ± SEM (n = 8). *Significant differences are indicated by ****P < 0.0001 and **P < 0.0054. The following abbreviations are used throughout the figure: **(A)** POMC (pro-opiomelanocortin). **(B)** MC1R (melanocortin receptor 1). **(C)** MC4R (melanocortin receptor 4). **(D)** AgRP (agouti-related peptide). **(E)** NPY (neuropeptide Y). **(F)** and NPY4R (neuropeptide Y receptor 4). **(G)** NPY5R (neuropeptide Y receptor 5).

**Figure 8:**
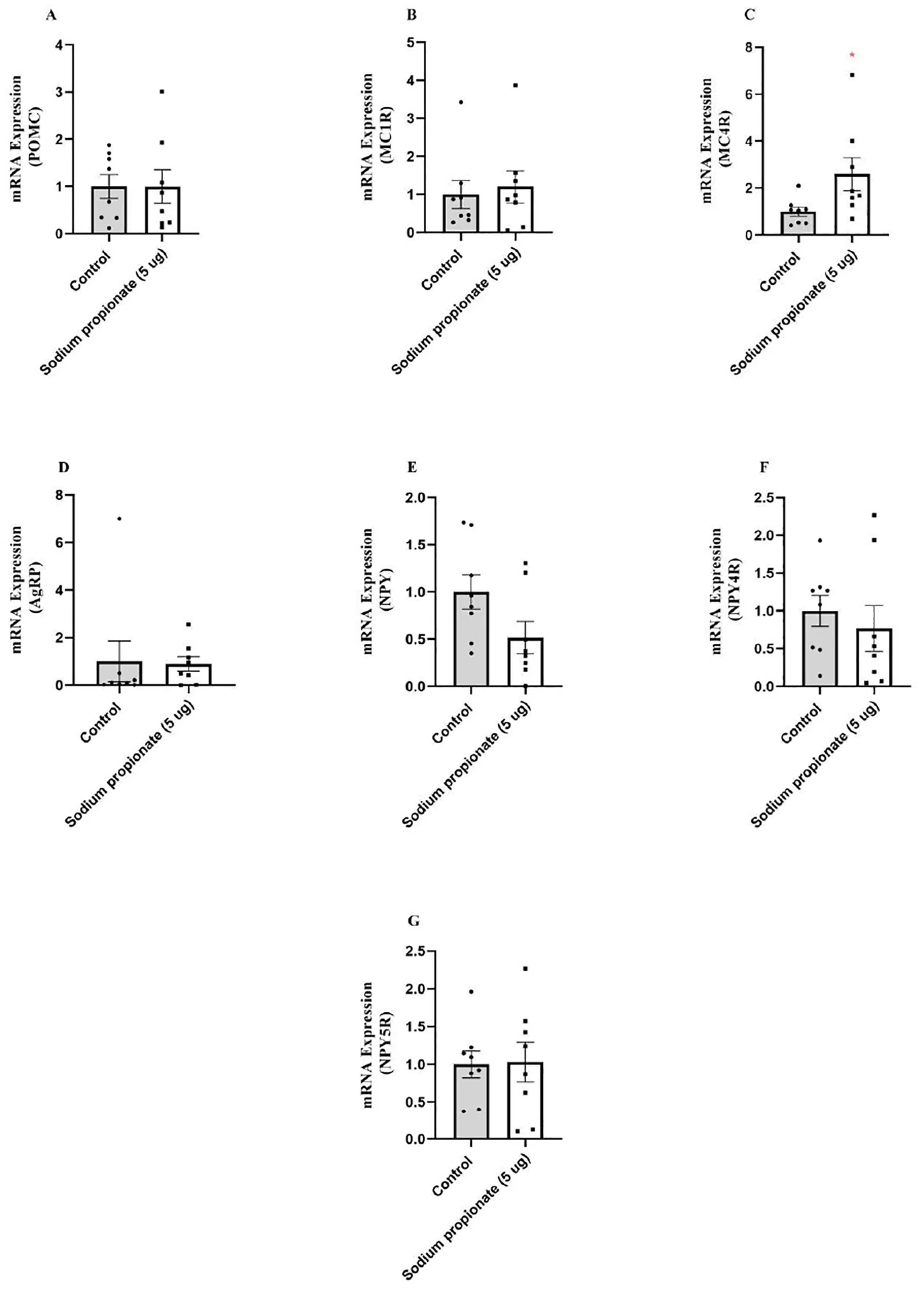
The impact of centrally delivered sodium propionate (5 mg) on the expression of hypothalamic genes involved in appetite regulation was examined in neonatal layer-type chicks. The data are presented as mean ± SEM (n = 8). *Significant differences are indicated by *P < 0.0459. The following abbreviations are used throughout the figure: **(A)** POMC (proopiomelanocortin). **(B)** MC1R (melanocortin receptor 1). **(C)** MC4R (melanocortin receptor 4). **(D)** AgRP (agouti-related peptide). **(E)** NPY (neuropeptide Y). **(F)** and NPY4R (neuropeptide Y receptor 4). **(G)** NPY5R (neuropeptide Y receptor 5).

## 4 Discussion

For the first time, this study shows that central injection of SCFAs affects feed intake and genes involved in appetite regulation in chicks. It’s interesting to note that our research used a specific method to test how central SCFA signaling affects the body. By using ICV administration, we were able to minimize the impact of the natural bacteria in our system on the results. However, since there’s a two-way conversation between the brain and other parts of the body, the changes we saw in the hypothalamus might also be due to secondary effects from other areas after the central administration. This means that while we were focusing on the central effects, the peripheral parts of the body could still be influencing the outcomes.

Understanding the mechanisms that regulate appetite is increasingly important, particularly in light of the growing human population and the rising demand for animal-derived products (Motaghi et al., 2021). Previous studies have examined appetite regulation in mammals (Frost et al., 2014); however, because hypothalamic control of food intake differs between mammals and chicks (Zendehdel and Hassanpour), it is also important to clarify how appetite is regulated in birds (Richards and Proszkowiec-Weglarz, 2007).

Studies in mammals have shown that SCFAs, especially acetate, influence hypothalamic neurons (Frost et al., 2014), which play a central role in the regulation of feed intake (Yousefvand and Hamidi, 2019). However, the study in mammals demonstrated that the effect of acetate on central appetite regulation was observed following peripheral administration (Frost et al., 2014). In poultry, SCFAs stimulate intestinal development in broilers and reduce the severity of necrotic enteritis (Liao et al., 2020; Gómez-Osorio et al., 2021). SCFAs have also been reported to improve eggshell quality and hatchability in layer chickens (Sengor et al., 2007) and to increase meat quality in broilers by lowering cholesterol and fat concentrations in the blood (which may contribute to improved meat quality) (Khatibjoo et al., 2018). However, the direct central effects of SCFAs on feed intake and hypothalamic appetite-related neuropeptide expression in chicks have not yet been clarified. In the present study, different SCFAs produced distinct effects on feed intake and on the mRNA levels of appetite-related neuropeptides in the hypothalamus.

Administration of sodium acetate (0.25 μg) activated orexigenic signaling pathways and stimulated feed intake; however, a reduction in feed intake has been reported in mice (Frost et al., 2014). By using 11C-acetate and pet-CT, it is demonstrated that acetate has a direct role in central appetite regulation of mice (Frost et al., 2014). This discrepancy shows that the central role of acetate in appetite regulation may differ between birds and mammals. This speculation, however, should be further investigated.

Following sodium acetate administration, hypothalamic *NPY5R* mRNA expression increased, supporting the established role of NPY as a key orexigenic peptide in the hypothalamus across vertebrate species (Nguyen et al., 2011; Yousefvand et al., 2019; Rahmani et al., 2021). Previous studies have shown that NPY increases feed intake in chicks, whereas its blockade reduces feed consumption (Chen G. et al., 2016). In the current study, our findings indicate that sodium acetate may stimulate feed intake through NPY receptor-related signaling pathways. The significant increase in *NPY5R* expression, together with the absence of changes in anorexigenic neuropeptides, indicates that sodium acetate may regulate feed intake mainly through the NPY5R pathway. The increased expression of *NPY5R* without a significant change in *NPY* expression may indicate that receptor availability enhances the responsiveness of hypothalamic neurons to existing NPY signaling.The hypothalamus serves as a central integrator of nutritional, hormonal, and metabolic information and regulates not only feeding behavior but also whole-body energy homeostasis through neuroendocrine and autonomic pathways. Therefore, changes in hypothalamic appetite-related neuropeptides may have broader physiological consequences beyond short-term feed intake regulation. Alterations in NPY and POMC/MC4R signaling may influence energy expenditure, nutrient partitioning, glucose metabolism, lipid metabolism, and endocrine responses through interactions with peripheral organs, including the liver, adipose tissue, gastrointestinal tract, and endocrine glands. Although the present study focused on acute feeding responses and hypothalamic gene expression, these findings suggest that central SCFA signaling may contribute to the coordination of whole-body metabolic responses in chickens. Future studies evaluating energy expenditure, glucose homeostasis, lipid metabolism, and peripheral tissue responses will be necessary to determine the systemic consequences of SCFA-induced hypothalamic changes.

In the present study, sodium acetate also increased *AgRP* expression, although this effect was not statistically significant in the present study. Sodium butyrate (0.5 μg) increased feed intake in chicks, whereas previous studies in mice reported a reduction in feed consumption following butyrate administration (Li et al., 2017). However, in this previous study (Li et al., 2017), the acute effects of butyrate on appetite were evaluated in mice following an intragastric gavage or intravenous injection of butyrate. Hence, the indirect influence of butyrate on appetite cannot be excluded. These findings further indicate that central SCFA action may differ among species.

Our study shows that sodium butyrate increases *NPY* expression while simultaneously reducing *POMC* and *MC4R* expression. Thus, sodium butyrate-induced hyperphagia may be associated with enhanced orexigenic signaling and suppressed anorexigenic signaling. In the present study, the effects of SCFAs on feed intake were not strictly dose-dependent. For example, sodium butyrate increased feed intake at the intermediate dose but not at the lower or higher doses, suggesting that hypothalamic responses to SCFAs may vary depending on concentration. However, variability in biological response, limited statistical power, or complex central sensitivity to SCFAs may induce this non-monotonic response.

Central sodium propionate (5 μg) reduced feed intake and increased *MC4R* expression. Consistent with our study, a recent study - also reported that propionate decreases feed intake and alters appetite-related neuropeptides in pigs infused with sodium propionate through a fistula in the caecum (Zhang et al., 2022). However, studies based on peripheral exposure may not be directly comparable to our observations following ICV administration. The increase in *MC4R* expression without changes in *POMC* expression may indicate that sodium propionate enhances the sensitivity of the melanocortin signaling pathway rather than increasing the production of its ligand. Although both sodium butyrate and sodium propionate affected *MC4R* expression, they had opposite effects on feed intake, and the mechanisms underlying these differences in chickens remain to be determined. MC4R and POMC neurons produce α-MSH, a peptide that suppresses appetite and supports energy balance (Mountjoy, 2015). The findings of this study are consistent with our previous reports showing that the central melanocortin system and NPY participate in regulating feed intake in laying chickens (Ghashghaei et al., 2025a; Ghashghaei et al., 2025b). To identify a level capable of altering feed intake, different doses of sodium propionate were evaluated in the present experiments; future studies should evaluate multiple doses within a single experiment to allow direct comparison among treatments. To really confirm these findings, it would be best to test a wide range of sodium propionate doses all at once, rather than trying them out one by one until we see an effect. This approach would give us more robust evidence and a clearer picture of how sodium propionate works. By doing so, we can be more confident in our results and better understand the relationship between the dose and the observed effect.

In addition, when a higher dose reduces feed intake, it may be due to changes in behavior; for example, it may induce malaise, leading to reduced feeding rather than specific appetite regulation. However, behavioral parameters were not assessed in the present study. This limits interpretation of whether the effect is specific or non-specific.

In our study, we collected whole hypothalamic tissue and therefore could not assess the direct effects of short-chain fatty acids specifically on the ARC, which is a key center for appetite regulation. This limits interpretation of region-specific hypothalamic responses. Also, the lack of a consistent dose–response relationship suggests that future studies should investigate variability and possible non-linear responses in central sensitivity to SCFAs. When you give SCFAs directly into the brain, it’s hard to tell if the effects you see are from the SCFAs working directly on the brain or if they’re coming from other parts of the body responding to the SCFAs. The brain and the gut are connected in a two-way street, with signals going back and forth through nerves, hormones, and metabolism. So, even if you put SCFAs right into the brain, the gut and other parts of the body might still react and send signals back to the brain, which could be what’s causing the changes you see in the hypothalamus.

Furthermore, gene expression analysis was performed only between the control group and the group receiving the effective dose of short-chain fatty acids, which limits interpretation of dose-dependent molecular responses and limits understanding of dose-related changes in gene expression.

Early-life regulation of feeding affects growth and later production performance in poultry; therefore, we conducted this study in neonatal chicks and evaluated short-term feed intake. The acute experimental design allows assessment of direct central effects; however, longer-term studies are required to determine physiological and production relevance. This limits extrapolation to long-term physiological conditions. In conclusion, sodium acetate, sodium butyrate, and sodium propionate have different effects on feed intake and alter the expression of both hypothalamic orexigenic and anorexigenic genes in chickens, accompanied by increased expression of the orexigenic neuropeptide *NPY* and decreased expression of the anorexigenic genes *POMC* and *MC4R.* These findings do not represent the physiological effects of SCFAs derived from the microbiota acting through the gut–brain axis. Rather, they demonstrate that SCFAs can modulate hypothalamic appetite-regulating pathways following central administration. However, as the gut–brain axis is bidirectional, the observed responses cannot be attributed solely to direct hypothalamic actions; indirect physiological effects may also be a factor. They contribute to the understanding of avian neuroendocrine control in feeding and may have practical implications for poultry production. A major strength of the present study is its use of a well-established intracerebroventricular administration model. The accuracy of the injections was confirmed using Evans Blue dye, providing a reliable experimental approach for evaluating central SCFA exposure and hypothalamic responses in neonatal chicks.

A previous study has demonstrated that acetate regulates feed intake through intracellular signaling proteins such as AMPK and ACC in mice (Frost et al., 2014); therefore, further investigation is required to determine whether similar signaling pathways mediate SCFA effects in chickens. Our study is limited to mRNA expression analysis and did not include protein-level validation of AMPK and ACC or neuronal activation markers (e.g., c-Fos); therefore, mechanistic conclusions should be considered preliminary. Despite these limitations, the present study provides novel evidence that central SCFA administration is associated with changes in hypothalamic appetite-regulating pathways in chicks and establishes a foundation for future mechanistic investigations.

## Data Availability

The original contributions presented in the study are included in the article, further inquiries can be directed to the corresponding author.

## References

[B1] Badri M. Alimohammadi S. Zendehdel M. Hassanpour S. (2025). Involvement of central opioid and melanocortin receptors in spexin-induced hypophagia following intracerebroventricular injection in neonatal broiler chicks. BMC Vet. Res. 21, 110. doi: 10.1186/s12917-025-04564-8 40011886 PMC11863689

[B2] Blouet C. Schwartz G. J. (2010). Hypothalamic nutrient sensing in the control of energy homeostasis. Behav. Brain Res. 209, 1–12. doi: 10.1016/j.bbr.2009.12.024 20035790

[B3] Byrne C. S. Chambers E. S. Morrison D. J. Frost G. S. (2015). The role of short chain fatty acids in appetite regulation and energy homeostasis. Int. J. Obes. 39, 1331–1338. doi: 10.1038/ijo.2015.84 25971927 PMC4564526

[B4] Camilleri M. J. (2015). Peripheral mechanisms in appetite regulation. Gastroenterology 148, 1219–1233. doi: 10.1053/j.gastro.2014.09.016 25241326 PMC4369188

[B5] Cao K.-X. Deng Z.-C. Li S.-J. Yi D. He X. Yang X.-J. . (2024). Poultry nutrition: achievement, challenge, and strategy. J. Nutr. 154, 3554–3565. doi: 10.1016/j.tjnut.2024.10.030 39424066

[B6] Chen G. Yang F. Wu T. Jiang J. Zhou W. (2016). The stimulatory effect of cerebral intraventricular injection of cNPY on precocial feeding behavior in neonatal chicks (Gallus domesticus). PloS One 11, e0153342. doi: 10.1371/journal.pone.0153342 27055273 PMC4824459

[B7] Chen Y. Lin Y.-C. Zimmerman C. A. Essner R. A. Knight Z. A. (2016). Hunger neurons drive feeding through a sustained, positive reinforcement signal. Elife 5, e18640. doi: 10.7554/elife.18640 27554486 PMC5016090

[B8] Davis J. L. Masuoka D. T. Gerbrandt L. K. Cherkin A. (1979). Autoradiographic distribution of L-proline in chicks after intracerebral injection. Physiol. Behav. 22, 693–695. doi: 10.1016/0031-9384(79)90233-6 482410

[B9] Forte N. Marfella B. Nicois A. Palomba L. Paris D. Motta A. . (2024). The short-chain fatty acid acetate modulates orexin/hypocretin neurons: a novel mechanism in gut-brain axis regulation of energy homeostasis and feeding. Biochem. Pharmacol. 226, 116383. doi: 10.1016/j.bcp.2024.116383 38908530

[B10] Frost G. S. Sleeth M. L. Sahuri-Arisoylu M. Lizarbe B. Cerdán S. Brody L. . (2014). The short-chain fatty acid acetate reduces appetite via a central homeostatic mechanism. Nat. Commun. 5, 3611. doi: 10.1038/ncomms4611 24781306 PMC4015327

[B11] Fu Y. (2025). Regulation of feeding behavior and body weight by orexigenic neurons in the arcuate nucleus. J. Obes. Metab. Syndr. 34, 213. doi: 10.7570/jomes25059 40707058 PMC12318705

[B12] Furuse M. Matsumoto M. Saito N. Sugahara K. Hasegawa S. (1997). The central corticotropin-releasing factor and glucagon-like peptide-1 in food intake of the neonatal chick. Eur. J. Pharmacol. 339, 211–214. doi: 10.1016/s0014-2999(97)01391-5 9473137

[B13] Ghashghaei E. Mijiyawa A. Wang M. Zendehdel M. Lin H. (2025a). Central effects of bile acids on feed intake and mRNA expression of appetite-related neuropeptides in layer-type chicks. Poul Sci. 104, 106105. doi: 10.1016/j.psj.2025.106105 41265140 PMC12666714

[B14] Ghashghaei E. Wang M. Mijiyawa A. Lin H. (2025b). Effects of amino acids methionine, lysine, and taurine on feed intake and mRNA expression of appetite-related neuropeptides in layer-type chicks. Poult. Sci. 104, 105586. doi: 10.1016/j.psj.2025.105586 40743628 PMC12336831

[B15] Ghashghaei E. Yuan X. Wang M. Liu M. Wang X. Jiao H. . (2025c). Effect of methionine replacement with taurine on the performance and antioxidative capacity of broiler. Poult. Sci. 104. doi: 10.1016/j.psj.2025.105505 40609327 PMC12272128

[B16] Ghashghayi E. Zendehdel M. Khodadadi M. Rahmani B. (2022). Central dopaminergic, serotoninergic, as well as GABAergic systems mediate NMU-induced hypophagia in newborn chicken. Int. J. Neurosci. 134, 353–363. doi: 10.1080/00207454.2022.2102980 35901030

[B17] Gómez-Osorio L. M. Yepes-Medina V. Ballou A. Parini M. Angel R. (2021). Short and medium chain fatty acids and their derivatives as a natural strategy in the control of necrotic enteritis and microbial homeostasis in broiler chickens. Front. Vet. Sci. 8. doi: 10.3389/fvets.2021.773372 34970616 PMC8712453

[B18] Hill J. W. (2010). Gene expression and the control of food intake by hypothalamic POMC/CART neurons. Open Neuroendocrinol. J. 3, 21. PMC5201111 28042349

[B19] Jiao A. Yu B. He J. Yu J. Zheng P. Luo Y. . (2020). Short chain fatty acids could prevent fat deposition in pigs via regulating related hormones and genes. Food Funct. 11, 1845–1855. doi: 10.1039/c9fo02585e 32067021

[B20] Khatibjoo A. Mahmoodi M. Fattahnia F. Akbari-Gharaei M. Shokri A. Soltani S. M. (2018). Effects of dietary short- and medium-chain fatty acids on performance, carcass traits, jejunum morphology, and serum parameters of broiler chickens. J. Appl. Anim. Res. 46, 492–498. doi: 10.1080/09712119.2017.134574137339054

[B21] Lenard N. R. Berthoud H.-R. (2008). Central and peripheral regulation of food intake and physical activity: pathways and genes. Obesity 16, S11–S22. doi: 10.1038/oby.2008.511 19190620 PMC2687326

[B22] Li Z. Yi C.-X. Katiraei S. Kooijman S. Zhou E. Chung C. K. . (2017). Butyrate reduces appetite and activates brown adipose tissue via the gut-brain neural circuit. Gut 67, 1269–1279. doi: 10.1136/gutjnl-2017-314050 29101261

[B23] Liao X. Shao Y. Sun G. Yang Y. Zhang L. Guo Y. . (2020). The relationship among gut microbiota, short-chain fatty acids, and intestinal morphology of growing and healthy broilers. Poult. Sci. 99, 5883–5895. doi: 10.1016/j.psj.2020.08.033 33142506 PMC7647869

[B24] Lu J. Wang Q. Wang K. Ma M. Wang X. Guo J. . (2023). Effects of energy restriction during growing phase on the productive performance of Hyline Brown laying hens aged 6 to 72 wk. Poult. Sci. 102, 102942. doi: 10.1016/j.psj.2023.102942 37566966 PMC10432841

[B25] Mahdavi K. Zendehdel M. Zarei H. (2024). Neuropeptide Y: an undisputed central simulator of avian appetite. J. Poult. Scie Avian Dis. 2, 1–8. doi: 10.61838/kman.jpsad.2.3.1

[B26] Mitchell R. W. On N. H. Del Bigio M. R. Miller D. W. Hatch G. M. (2011). Fatty acid transport protein expression in human brain and potential role in fatty acid transport across human brain microvessel endothelial cells. J. Neurochem. 117, 735–746. doi: 10.1111/j.1471-4159.2011.07245.x 21395585

[B27] Motaghi S. Jonaidi H. Bashiri A. Gooshki S. N. (2021). Purinergic regulation of food and fat intakes in broiler’s central nervous system. Iran. J. Vet. Med. 404–410.

[B28] Mountjoy K. (2015). Pro‐opiomelanocortin (POMC) neurones, POMC‐derived peptides, melanocortin receptors and obesity: how understanding of this system has changed over the last decade. J. Neuroendocrinol. 27, 406–418. doi: 10.1111/jne.12285 25872650

[B29] Newmyer B. A. Nandar W. Webster R. I. Gilbert E. Siegel P. B. Cline M. A. (2013). Neuropeptide Y is associated with changes in appetite-associated hypothalamic nuclei but not food intake in a hypophagic avian model. Behav. Brain Res. 236, 327–331. doi: 10.1016/j.bbr.2012.08.015 22921928

[B30] Nguyen A. D. Herzog H. Sainsbury A. (2011). Neuropeptide Y and peptide YY: important regulators of energy metabolism. Curr. Opin. Endocrinol. Diabetes Obes. 18, 56–60. doi: 10.1097/med.0b013e3283422f0a 21157324

[B31] Pérez-Bonilla A. Novoa S. García J. Mohiti-Asli M. Frikha M. Mateos G. (2012). Effects of energy concentration of the diet on productive performance and egg quality of brown egg-laying hens differing in initial body weight. Poult. Sci. 9, 3156–3166. doi: 10.3382/ps.2012-02526 23155026

[B32] Rahmani B. Ghashghayi E. Zendehdel M. Khodadadi M. Hamidi B. (2021). The crosstalk between brain mediators regulating food intake behavior in birds: a review. Int. J. Pept. Res. Ther. 27, 2349–2370. doi: 10.1007/s10989-021-10257-130311153

[B33] Ramser A. Dridi S. (2022). Avian orexin: feed intake regulator or something else? Vet. Sci. 9, 112. doi: 10.3390/vetsci9030112 35324840 PMC8950792

[B34] Richards M. Proszkowiec-Weglarz M. (2007). Mechanisms regulating feed intake, energy expenditure, and body weight in poultry. Poul Sci. 86, 1478–1490. doi: 10.1093/ps/86.7.1478 17575199

[B35] Safikhani A. Zendehdel M. Khodadadi M. Rahmani B. Ghashghayi E. Mahdavi K. (2023). Hypophagia induced by intracerebroventricular injection of apelin-13 is mediated via CRF1/CRF2 and MC3/MC4 receptors in neonatal broiler chicken. Behav. Brain Res. 452, 114536. doi: 10.1016/j.bbr.2023.114536 37295613

[B36] Saneyasu T. (2024). Recent research on mechanisms of feeding regulation in chicks. J. Poul Sci. 61, 2024012. doi: 10.2141/jpsa.2024012 38681189 PMC11039390

[B37] Sengor E. Yardımcı M. E. Cetingul S. Bayram I. Sahin H. Dogan I. (2007). Effects of short chain fatty acid (SCFA) supplementation on performance and egg characteristics of old breeder hens : : short communication. S. Afr. J. Anim. Sci. 37, 158–163. doi: 10.4314/sajas.v37i3.4086

[B38] Ueno H. Nakazato M. (2016). Mechanistic relationship between the vagal afferent pathway, central nervous system and peripheral organs in appetite regulation. J. Diabetes Investiga 7, 812–818. doi: 10.1111/jdi.12492 27180615 PMC5089941

[B39] Vadder F. D. Kovatcheva-Datchary P. Goncalves D. Vinera J. Zitoun C. Duchampt A. . (2014). Microbiota-generated metabolites promote metabolic benefits via gut-brain neural circuits. Cell. 156, 84–96. doi: 10.1016/j.cell.2013.12.016 24412651

[B40] Van Tienhoven A. Juhász L. P. (1962). The chicken telencephalon, diencephalon and mesencephalon in stereotaxic coordinates. J. Comp. Neurol. 118, 185–197. doi: 10.1002/cne.901180205 13924637

[B41] Vlaicu P. A. Untea A. E. Oancea A. G. (2024). Sustainable poultry feeding strategies for achieving zero hunger and enhancing food quality. Agriculture 14, 1811. doi: 10.3390/agriculture1410181130654563

[B42] Yousefvand S. Hamidi F. (2019). Role of paraventricular nucleus in regulation of feeding behaviour and the design of intranuclear neuronal pathway communications. Int. J. Pept. Res. Ther. 26, 1231–1242. doi: 10.1007/s10989-019-09928-x30311153

[B43] Yousefvand S. Hamidi F. Zendehdel M. Parham A. (2019). Survey the effect of insulin on modulating feed intake via NPY receptors in 5-day-old chickens. Int. J. Pept. Res. Ther. 26, 467–476. doi: 10.1007/s10989-019-09852-030311153

[B44] Zarei H. Kiaee G. Zendehdel M. (2025). NPY1 and MC3/MC4 receptors mediate BDNF-induced hypophagia in 5-day-old chickens. J. Poult. Sci. Avian Dis. 3, 74–83. doi: 10.61838/kman.jpsad.3.1.7

[B45] Zendehdel M. Ghashghayi E. Hassanpour S. Baghbanzadeh A. Jonaidi H. (2016). Interaction between opioidergic and dopaminergic systems on food intake in neonatal layer type chicken. Int. J. Pept. Res. Ther. 22, 83–92. doi: 10.1007/s10989-015-9486-430311153

[B46] Zendehdel M. Hassanpour S. (2014). Central regulation of food intake in mammals and birds. Neurotransmitter. 1, e251. doi: 10.14800/nt.251

[B47] Zendehdel M. Sardari F. Hassanpour S. Rahnema M. Adeli A. Ghashghayi E. (2017). Serotonin-induced hypophagia is mediated via α2 and β2 adrenergic receptors in neonatal layer-type chickens. Br. Poult. Sci. 58, 298–304. doi: 10.1080/00071668.2017.1278626 28362179

[B48] Zhang Y. Li X. Huang G. Wang H. Chen H. Su Y. . (2022). Propionate stimulates the secretion of satiety hormones and reduces acute appetite in a cecal fistula pig model. Anim. Nutr. 10, 390–398. doi: 10.1016/j.aninu.2022.06.003 35949198 PMC9356018

[B49] Zhao J. P. Jiao H. C. Song Z. Lin H. (2009). Effects of L-arginine supplementation on glucose and nitric oxide (NO) levels and activity of NO synthase in corticosterone-challenged broiler chickens (Gallus gallus). Comp. Biochem. Physiol. C. Toxicol. Pharmacol. 150, 474–480. doi: 10.1016/j.cbpc.2009.07.003 19615465

